# Design of Automatic Tool Replacement Mechanism for Laparoscopic Surgical Robot Arm for Solo Surgery

**DOI:** 10.1002/rcs.70106

**Published:** 2025-09-03

**Authors:** Daehwan Ko, Yeonkyoung Kim, Hongseok Lim, Sungmin Kim

**Affiliations:** ^1^ Research Institute for Commercialization of Biomedical Convergence Technology Dongguk University Goyang South Korea; ^2^ Department of Regulatory Science for Medical Device Dongguk University Seoul South Korea; ^3^ Department of Mechanical Engineering Sogang University Seoul South Korea; ^4^ Department of Biomedical Engineering Dongguk University Goyang South Korea; ^5^ Department of Medical Device and Healthcare Dongguk University Seoul South Korea

**Keywords:** automatic tool replacement, FAST diagram, laparoscopic surgical robot, solo surgery

## Abstract

**Background:**

Laparoscopic robotic surgery requires intraoperative tool replacement owing to the limited number of surgical tools that can be used simultaneously. Currently, this process is performed by a surgical assistant. However, automatic tool replacement is essential for surgeons when operating alone.

**Methods:**

An initial design was constructed by analysing the FAST diagram of the surgical tool replacement process. It was then modified to arrive at the final design by considering the driving range of the robot arm.

**Results:**

Based on the final design, both simulation and robot arm manufacturing were performed and validated. The results showed that the posture could be maintained during tool replacement, and the entire tool replacement process could be performed in 15 s.

**Conclusions:**

The mechanism developed for the automatic replacement of surgical tools is expected to address the shortage of surgical staff and skill level of surgical assistants.

## 1 Introduction

Laparoscopic surgery is a minimally invasive procedure performed on the abdomen and involves the insertion of surgical tools through three to four small incisions. This minimally invasive procedure is preferred by many patients because it is less painful to the patient, reduces the risk of postoperative infections, and shortens the recovery time [1, 2]. However, from the surgeon's perspective, laparoscopic surgery presents many challenges compared with open surgery, including difficulties in visual feedback and the need to manipulate complex instruments [3, 4, 5]. Researchers have developed techniques to mitigate these difficulties, among which laparoscopic surgical robots have been effective in addressing these challenges and improving the ability of the surgeons to operate [5, 6, 7].

The presence of one or more surgical assistants is essential in current laparoscopic robotic surgeries. The surgical assistant provides support to the surgeon by manipulating the camera to ensure the surgeon's vision and replacing tools on the robotic arm. Studies have shown that the skill level of these surgical assistants can make a significant difference during surgeries [8, 9]. The role of a surgical assistant may appear simple; however, they play a crucial role in the surgical process and must be in sync with the surgeon to ensure a successful surgery in a short time. Some recent studies have reported on robotic surgical assistants taking over this role to reduce human errors [10, 11].

Bauzano et al. found that replacing surgical assistants with robotic systems allows surgeons to focus on the surgical procedure, and that robots are easier to handle and require less training time than human assistants [12]. Bucher et al. showed that in laparoscopic surgeries, robotic systems can act as surgical assistants, thus facilitating solo surgeries [13]. Kim et al. showed that when a surgical assistant was replaced by a robot and a single person controlled both the surgical robot and surgical assistant simultaneously, collisions between the robotic arm and assistive devices could be prevented, resulting in a smooth operation [14]. Many of these studies addressed the replacement of a surgical assistant for solo surgeries and therefore proposed methods to control the robot without imposing an additional burden on the surgeon [13, 15].

The aforementioned studies aimed to replace the surgical assistant with a robotic system; however, to fully replace a surgical assistant, automatically implementing one of the roles of the surgical assistant, namely handling the surgical tools, is essential. The challenge for surgical robotic systems is positioning the surgical tools in the desired position after the tool replacement [16]. Therefore, all parts except the tool replacement adaptor should be motionless, and the tool replacement adaptor should implement a linear motion to quickly position the tool in the same position as the one before it was replaced. There are systems for replacing ophthalmic surgical instruments that meet these conditions, but they are mechanisms designed for very small and light ophthalmic surgical instruments [17]. Therefore, if we use the same mechanism to create a laparoscopic surgical instrument replacement system, we will face the problem of excessive driving force and excessive inertial momentum caused by the large and heavy laparoscopic surgical instruments. This necessitates the development of new mechanisms to automate one of the critical tasks of surgical assistants, namely, tool replacement in surgical robotic systems.

**FIGURE 1:**
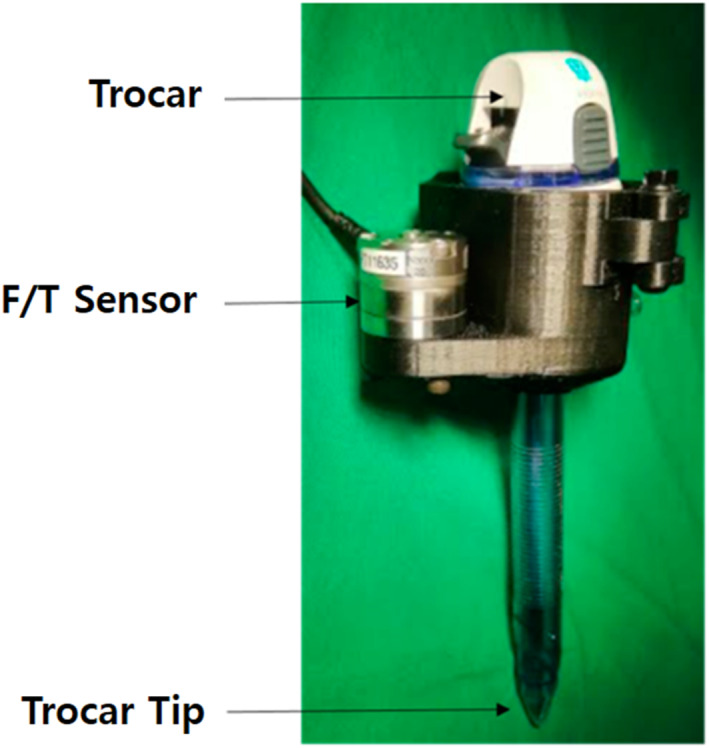
Trocar used for laparoscopic surgery (adapted and reprinted with permission from ref. [18]. Licence: https://creativecommons.org/licenses/by/4.0).

Automatic tool replacement mechanisms for surgical robotic systems must reflect the characteristics of laparoscopic robotic surgery. Laparoscopic robotic surgery is different from an open surgery in that the former uses a surgical instrument called trocar [Figure 1] [18]. A trocar consists of a casing tube and piercing cone. The piercing cone perforates the surgical site and is then removed, and the casing tube provides an entry for the surgical tools into the abdomen through itself [19]. As the casing tube of the trocar is inserted into the human body and fixed, and the surgical tool can enter the human body only through the casing tube, the angle of insertion of the surgical tool is limited. Therefore, the existing robotic surgical tool replacement uses a straight‐rail movement, and it is desirable for the new mechanism to be implemented based on the straight‐rail movement. Moreover, in robotic laparoscopic surgery, no entry methods other than those provided through the trocar exist. Therefore, specialised methods exist to create the angle of the surgical tools for surgery without dilating or damaging the entry. Most of the methods, currently in use, are based on the remote centre motion (RCM), which connects multiple links and uses the motion from the rotational centre of the links to cause them to rotate about an imaginary centre of rotation. Thus, during a surgery, all instrument motions are limited to the trocar's centre of rotation (RCM point) [20, 21]. As a result, the movement of the arm away from the RCM point would be very inefficient, even during an intraoperative tool replacement. Therefore, an automated robotic tool replacement system should be designed such that the arm axis remains fixed to the RCM point on the trocar. However, many of the automatic tool replacement mechanisms, currently being researched in the field, require the robot arm to move and actively retrieve the tool from the rack, which does not satisfy the requirement that the tool replacement must be performed while being fixed to the RCM point [22, 23, 24].

Therefore, to develop a new tool replacement mechanism satisfying the aforementioned requirements, this study analysed the tool replacement process of an existing surgical robot through a functional analysis method and constructed a FAST (Function Analysis and System Technique) diagram, as described in Section 2. Based on this, we added the necessary functions considering the safety aspects and developed an automated tool replacement mechanism for a surgical robot arm. In Section 3, we describe the development of a detailed power‐transmission mechanism for the actual drive and present the design of an automated surgical tool changer. We also performed kinematic drive simulations on the derived design model to verify its drivability and make design modifications to improve its performance. For the final design, we built a robotic system for the automated tool replacement of the surgical tools; the validation results from an actual operation are presented in Section 3, and the analysis of the results are presented in Section 4. Finally, Section 5 presents the conclusions of this research.

The developed and validated laparoscopic surgical robotic automatic tool replacement mechanism is expected to contribute to the implementation of solo‐robotic surgery systems by automating the tasks of surgical assistants in conventional robotic surgeries.

## 2 Materials and Method

This study did not involve any human participants, human data, or human tissue. As such, ethical approval was not required.

As mentioned in the introduction, the mechanism for automatic surgical tool replacement has some requirements.

- The tool replacement motion should be implemented as a linear motion in order to insert the tool within the insertion angle limited by the casing tube.
- Tool replacement should be possible without the robot arm moving (fixed to the RCM Point).
- Instrument design should eliminate the possibility of patient contact during surgery.
- Three quantitative goals:
  — The time required to replace surgical instruments should be within 15 s.
  — The mass of the automatic surgical instrument replacement device should be less than 4 kg.
  — The overall size of the automatic surgical instrument replacement device should be similar to that of existing surgical robots.

Among the quantitative targets presented, the replacement time was determined based on the manual replacement time of surgical instruments, the replacement device mass was determined based on the load capacity of the surgical assistant robot arm, and the overall size was determined to be similar to that of the existing single surgical instrument mounting section in order to minimise the rotational moment of the replacement device and robot arm connection section and to ensure a safe distance from other equipment in the operating room.

**FIGURE 2:**
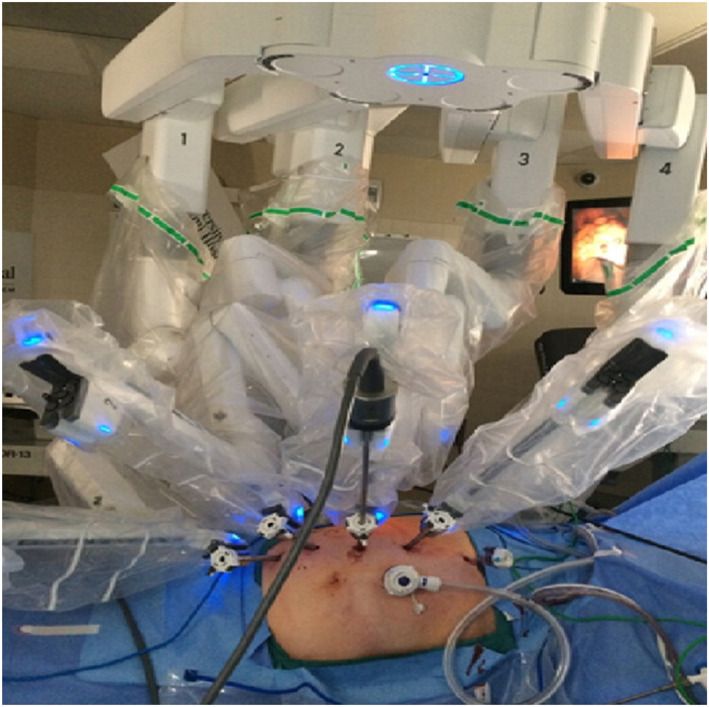
Da Vinci robot surgical system (adapted and reprinted with permission from ref. [25]. Licence: https://creativecommons.org/licenses/by/4.0).

**FIGURE 3:**
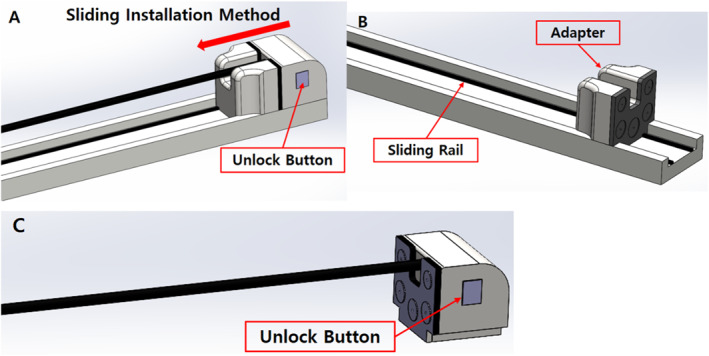
Tool replacement structure of the Da Vinci surgical robot. (A) Surgical tool combined with the adaptor. (B) Adaptor and sliding rail. (C) Surgical tool part.

Before developing the tool replacement mechanism for surgical robots, we analysed the most popular Da Vinci surgical robots used in hospitals to understand their basic requirements [Figure 2] [25]. In a Da Vinci surgical robotics system, the surgical tool replacement is performed in a sliding fashion. When removing a surgical tool, an unlock button is pressed and the surgical tool is pulled along the sliding rail to remove it; a new surgical tool is advanced along the sliding rail towards the adaptor to mount on the same; this completes the tool replacement [Figure 3]. The Da Vinci's tool replacement method involves a straight sliding, which satisfies the requirement for the surgical instrument insertion angle because the robot arm (slider) is fixed; furthermore, because a human replaces the tool, it also satisfies the requirement that the tool replacement be performed while fixed to the RCM point.

Because the existing surgical robot tool replacement system satisfies both requirements of the surgical instrument replacement mechanism mentioned in the introduction, we developed an automatic tool replacement system based on the existing method. Therefore, we maintained a straight slide frame to implement the straight‐sliding method, which is a characteristic of the tool replacement method of the existing surgical robots, and aimed to create a design, in which the surgical tool can be moved forward and backward only inside the slider. We also added a few degrees‐of‐freedom to enable the automatic tool replacement.

### 2.1 Functional Analysis of Surgical Tool Replacement

**FIGURE 4:**
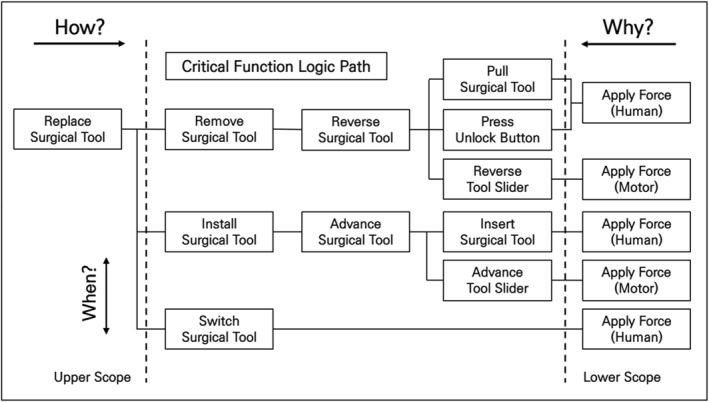
FAST diagram of the surgical tool replacement process.

To develop an automatic tool replacement mechanism for surgical tools, analysing the tool replacement process in existing surgical robots is crucial. Therefore, we did this using FAST, a technique that develops a graphical representation showing the logical relationships of the process based on ‘how?’ and ‘why?’ questions [Figure 4].

**FIGURE 5:**
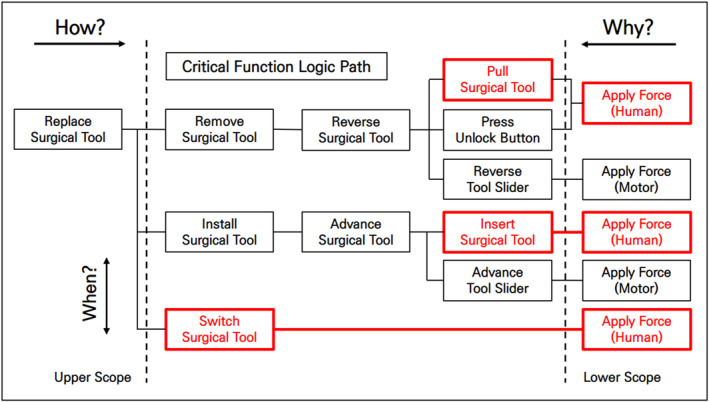
FAST diagram of the surgical tool replacement process (with parts to be modified).

The process of replacing the surgical tools has been divided into three main tasks: (1) removing the tool in use, (2) selecting and replacing a new tool, and (3) installing the new tool. Performing these tasks requires either motors or human power. Our analysis showed that four of the detailed tasks (pull surgical tool, press unlock button, insert surgical tool, switch surgical tool) required human intervention. Therefore, we needed to replace these tasks with motorised forces for automated tool replacement. In Figure 5, the areas that needed to be changed are marked in red on the FAST diagram.

The analysis showed that among the four tasks that required human intervention, the button‐pressing task could be directly replaced by a motor‐based operation. However, additional mechanisms are needed to automatically switch the surgical tools and to insert and pull the surgical tools. First, to switch the surgical tools automatically, multiple types of surgical tools must exist within the range of movement of the surgical robot. Automatic tool replacement devices in other fields use a robot that has a tool kit within its range of motion, and the tools are replaced in an automated manner as the robot moves. However, in the case of surgical robots, the tools need to maintain the RCM at all times; therefore, moving the entire robot arm to replace the tools during a surgery is very inefficient and potentially accident‐prone. For these reasons, automatic tool replacement should ideally take place when the surgical robot arm is stationary, and to accomplish the same, multiple surgical tools must be mounted on the surgical robot arm.

**FIGURE 6:**
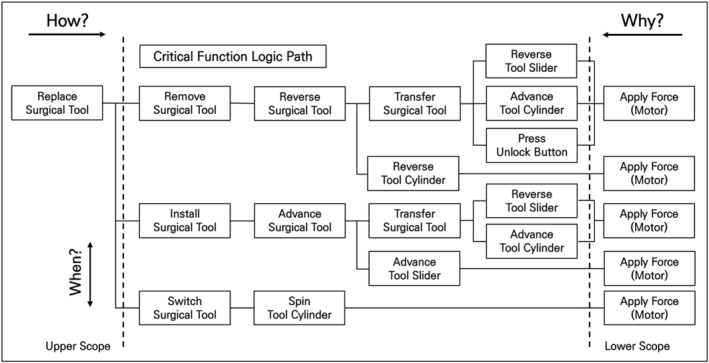
Final design FAST diagram of the surgical tool replacement process.

Mounting multiple surgical tools on a surgical robotic arm requires an additional mechanism. Here, we adopted a tool magazine mechanism from the literature tool magazine, which is essentially a platform for switching between different tools [26, 27]. Most tool magazines use rotation to switch between the tools, which works well with a system that replaces tools in the same state with a constant movement. Therefore, in this study, we introduced an additional rotating system for tool switching in a surgical robot, which allowed us to revise the replacement process and reorganise the fast diagram accordingly [Figure 6].

**FIGURE 7:**
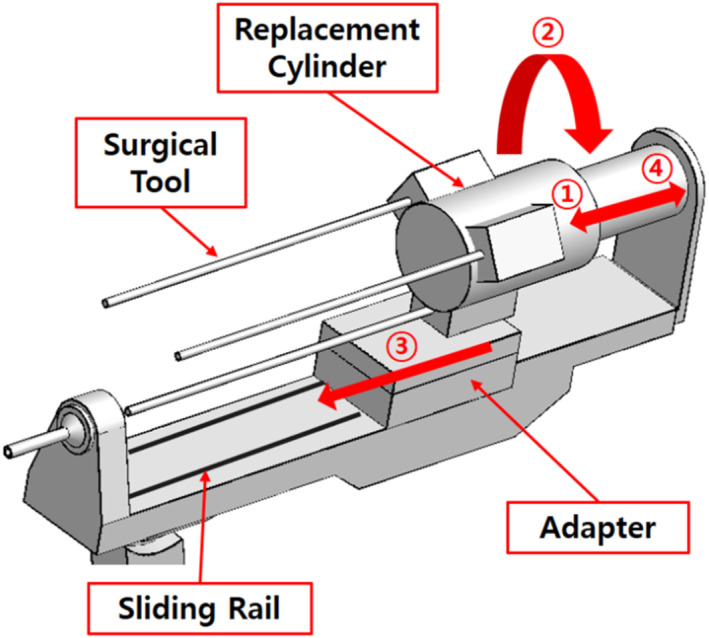
Initial (draft) design of tool replacement mechanism.

In this study, first an initial design of a surgical robot tool replacement mechanism was constructed based on the final fast diagram [Figure 7]. The new mechanism was designed to introduce a rotational system for tool switching and to re‐enter the patient's body through the trocar hole in a linear motion.

The mechanical component of the initial mechanism can be broadly divided into a slider, an adaptor, a replacement cylinder, and a surgical tool. The slider, which acts as the body of the surgical robot arm, contains rails that allow the adaptor to move back and forth. The adaptor is the part where the surgical tools are mounted; it moves on the sliding rails and is responsible for the forward and backward functions of the surgical tools. The rotating cylinder is located at the top behind the slider and is responsible for rotating and switching between the tools.

The initial mechanism is driven in four stages. In the first step, the adaptor with the tool is moved backward, and the replacement cylinder with the tool is moved forward. In the second step, the surgical tool on the adaptor is loaded onto the replacement cylinder, which rotates to switch the tools. In the third step, the surgical tool is mounted back onto the adaptor, which is advanced to reinsert the surgical tool back into the body. In the final step, the replacement cylinder reverses and returns to its original position, thus completing the tool replacement.

### 2.2 Design Modifications Based on Surgical Robot Arm Range of Motion

**FIGURE 8:**
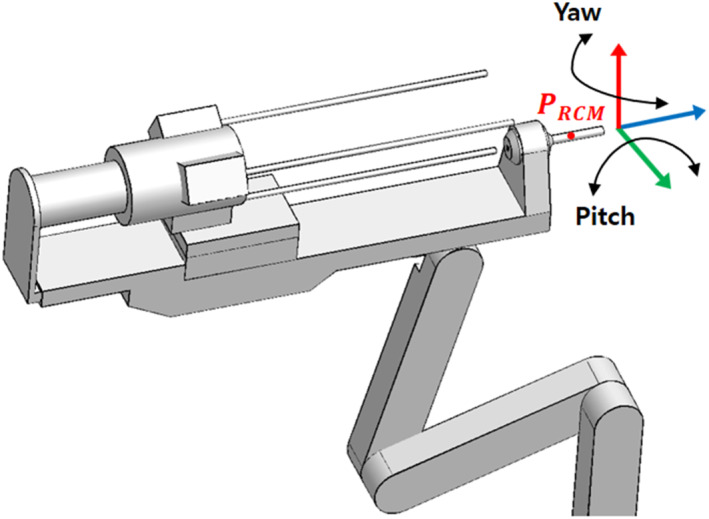
Yawing and pitching of surgical robot arm ($P_{RCM}$ – RCM Point).

In laparoscopic surgery, the procedure is performed through limited incisions; therefore, the tools rotate around the RCM point to achieve various positions required for surgery inside the abdomen. Surgical robots also perform rotational movements while maintaining the RCM point, which is the centre of rotation of the trocar. The RCM point remains fixed during surgery. In this case, yawing is defined as the left and right rotational motion, and pitching as the up and down rotational motion relative to the longitudinal axis of the surgical tool [Figure 8]. In this study, the range of motion of the surgical robot during a surgery was set to a maximum of ± 80° in the yaw direction and ± 60° in the pitch direction to reflect clinical requirements.

Because the surgical robot implements yawing and pitching motions with the RCM fixed, we can use this as the basis for configuring a contact safety zone to avoid contact with the patient and other parts during rotational motions. Assuming that the patient's body is positioned under a straight line perpendicular to the axis of the surgical tool based on the RCM, the contact safety zone is configured based on the rotational range of motion of the surgical robot. Based on the RCM point, we drew a line perpendicular to the patient's body and set a safe contact area where the surgical robot would not come into contact with the patient at its maximum range of motion: ± 80° in the yaw direction and ± 60° in the pitch direction. When performing actual surgery, the angle of the surgical tool replacement device also rotates accordingly; therefore, it is necessary to determine whether the replacement device is in contact with the patient within the specified rotation angle.

**FIGURE 9:**
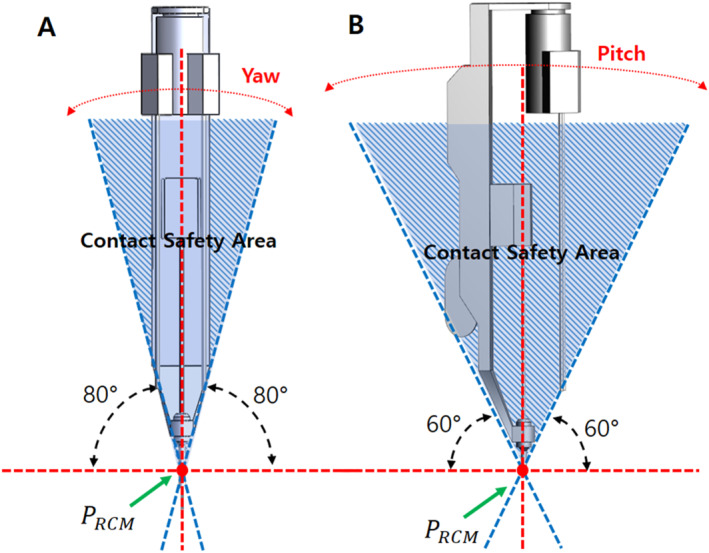
Contact safety area of the tool replacement mechanism after design amendments. (A) Considering yawing range. (B) Considering pitching range.

If a surgical robot has parts that protrude outside the contact safety zone, they might come in contact with the patient during rotational movements during the surgery; this could lead to a major mishap. To prevent this, we configured the contact safety zone based on the initial mechanism design of the surgical robot and attempted to configure a design that did not deviate significantly from the contact safety zone. Consequently, we changed the design of the slider parts [Figure 7] outside the contact safety zone in the initial design to allow all parts to fit inside the contact safety zone [Figure 9].

**FIGURE 10:**
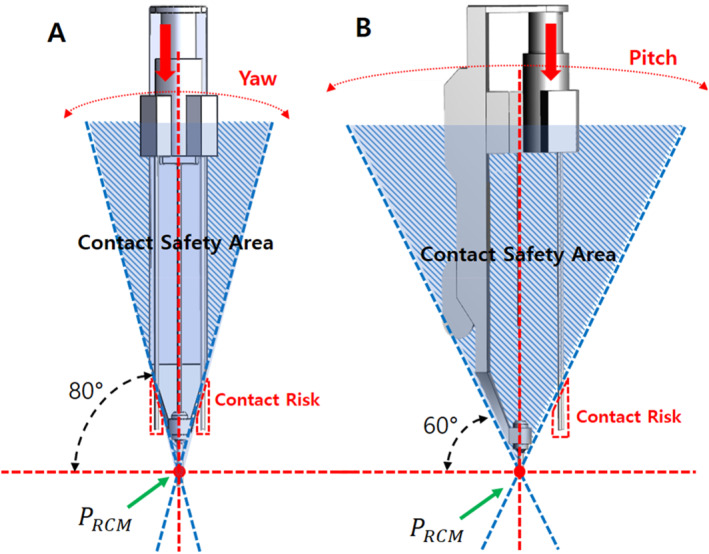
Out of contact safety area of surgical tool replacement mechanism after design amendment. (A) Considering yawing range. (B) Considering pitching range.

In a laparoscopic surgery, the movement in other parts is relatively large because the surgical tools move with the RCM point fixed. Because the contact safety zone configured in this study was based on the rotational range of motion, additionally considering the translational motion of the tools during the surgical procedure was necessary to ensure that they did not go outside the contact safety zone. As shown in Figure 9, the initial state of the revised instrument structure was within the safety zone for yawing and pitching motions [Figure 9]; however, during the process of mounting the surgical tool on the adaptor, the cylinder advanced, and the tip of the surgical tool was outside the contact safety zone, which could have led to a patient contact [Figure 10]. Therefore, a modification of the drive mechanism was required to ensure patient contact safety, as a design change to the instrument structure alone was not sufficient.

**FIGURE 11:**
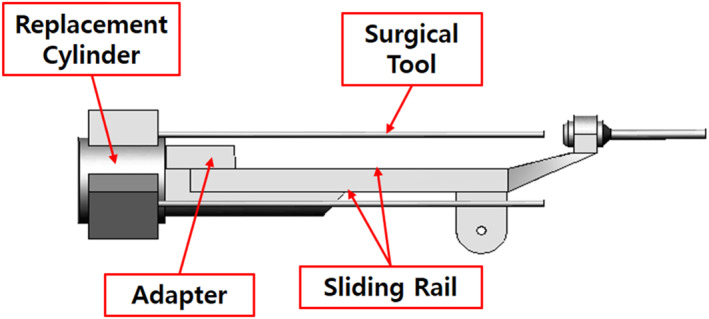
Structure of the final design of surgical robot arm.

**FIGURE 12:**
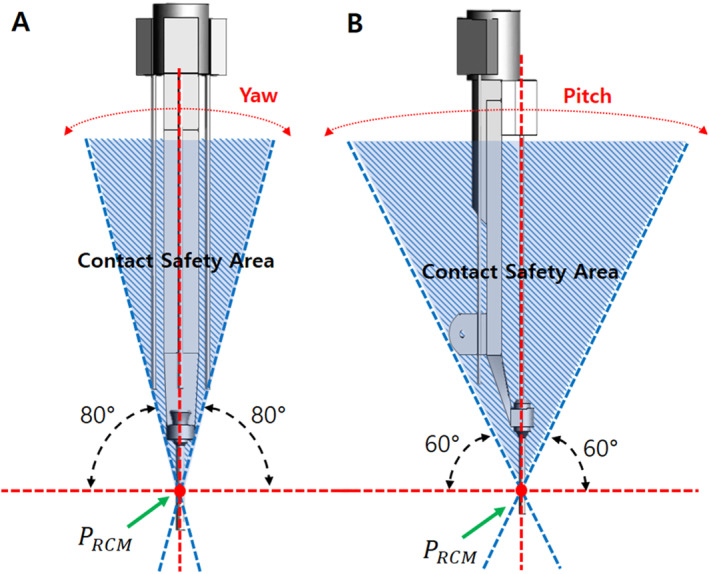
Contact safety area of surgical tool replacement mechanism in the final design. (A) Considering yawing range. (B) Considering pitching range.

**FIGURE 13:**
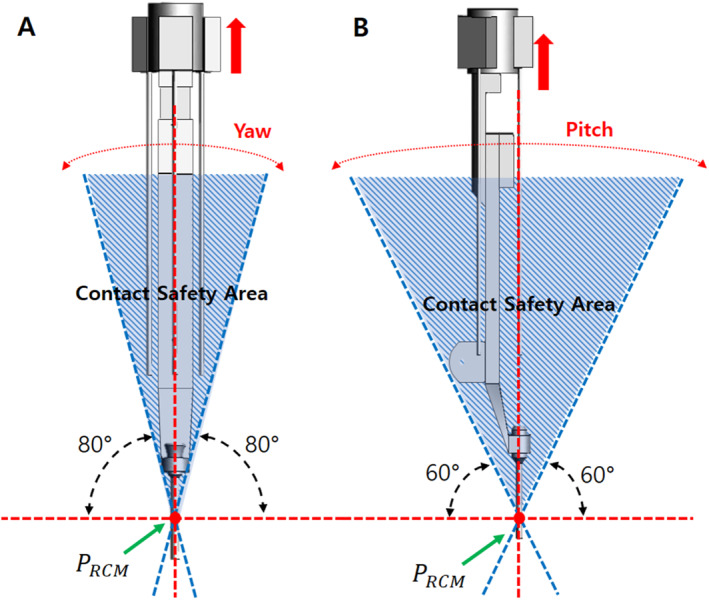
Contact safety area of surgical tool replacement mechanism in final design (replacement cylinder reversed). (A) Considering yawing range. (B) Considering pitching range.

To modify the drive mechanism, we proposed a modified structure in which the replacement cylinder is positioned behind the slider on the same line as that of the slider [Figure 11]. The modified mechanism consists of a sliding rail, an adaptor, a replacement cylinder, and surgical tools, and the overall structure is similar to the existing mechanisms. The adaptor is the part on which the surgical tool is mounted, and it moves on a sliding rail to perform forward and backward movements of the surgical tool. The replacement cylinder is loaded with tools and allows for the switching of surgical tools. The rails are located above and below the slider, with the top rail allowing the adaptor to move back and forth, and the bottom rail allowing the replacement cylinder to move likewise. The cylinder with the replacement tools is located on the same line as the slider; therefore, the storage location of the replacement tools is behind the slider, thereby eliminating the problem of leaving the contact safety zone when replacing surgical tools [Figures 12, 13].

**FIGURE 14:**
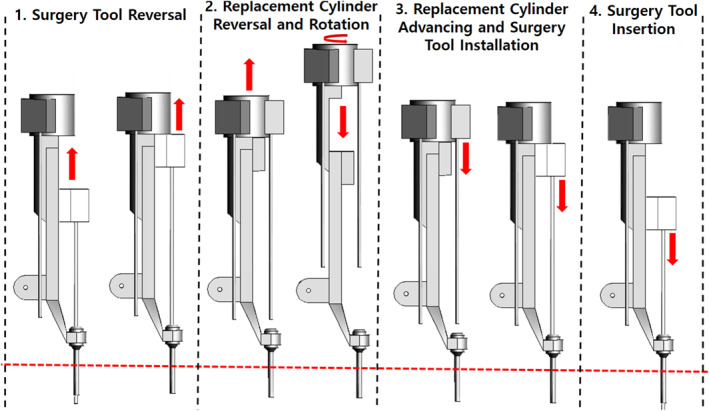
Tool replacement process of the final mechanism design.

The modified mechanism is operated in four steps, as shown in Figure 14. In the first step, the adaptor is reversed and moves in front of the surgical tool replacement cylinder. In the second step, the current tool is moved to the replacement cylinder, which moves backward and rotates to switch the surgical tool. In the third step, the replacement cylinder moves forward again, and the replaced surgical tool is mounted on the adaptor. In the final step, the adaptor advances to insert the surgical tool into the body, thereby finalising the tool change.

An additional improvement to the modified mechanism is that the moment generated when the robot arm rotates is minimised to increase the operating efficiency of the surgical robot. As shown in Figure 14, the overall length of the instrument body increases during the tool replacement; however, it is shortened after the replacement is complete; this allows us to reduce the moment applied to the rotary joint connected to the robot arm of the instrument body when the robot arm is driven. In the end, the modified mechanism model did not differ significantly from the existing surgical robot mechanisms, and by implementing the automatic replacement function, no significant differences in the space required were observed; based on this finding, we predicted that no interference with other devices would be encountered during an actual surgery.

## 3 Results

### 3.1 Design Details of the Automated Surgical Tool Replacement System

**FIGURE 15:**
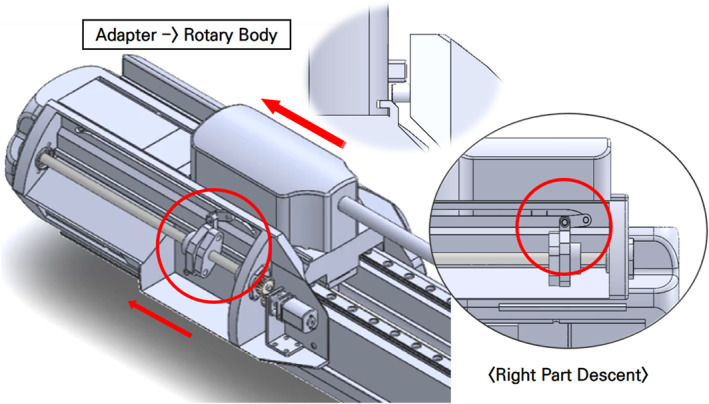
Principle of moving the surgical tool from adaptor to replacement cylinder. (In the case of the left side, the same mechanism is used to move the surgical instrument in the opposite direction).

The new tool replacement mechanism of the surgical robot requires perfect installation without wobbling, so that the subsequent surgical procedure can proceed without any problems. To ensure this, we designed an additional mechanism for installing and removing the adaptor. The surgical tool is pushed by pins on the left and right sides so that it moves along the rails to the adaptor installation (or removal) position. As shown in Figure 15, the pin on the right side of the surgical tool would be responsible for moving the surgical tool from the adaptor to the replacement cylinder after the removal, while that on the left side would be responsible for moving it from the replacement cylinder to the adaptor when the surgical tool was installed [Figure 15].

As shown in Figure 15, the travelling pins on both sides were designed to enter the dropout when not in use so that they do not interfere with the movement of the surgical tools. This means that when the right pin moved the surgical tool to the replacement cylinder, the left pin would enter the dropout, and when the left pin moved the surgical tool to the adaptor, the right pin would enter the dropout [Figure 15].

**FIGURE 16:**
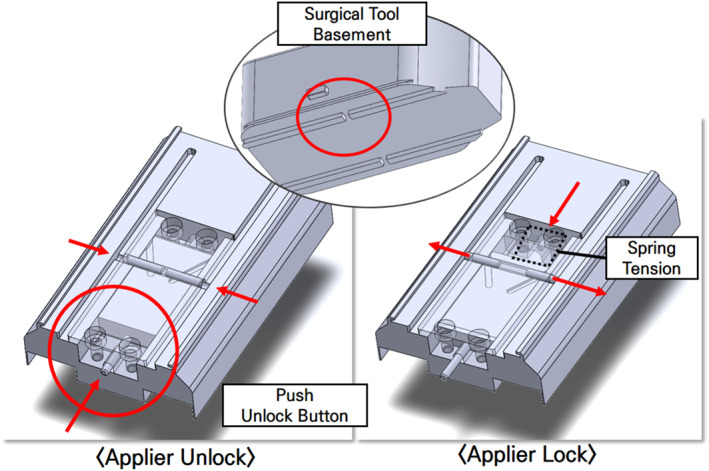
Fixed surgical tool adaptor: unlocking principle.

When the surgical tool was installed in the adaptor, it would use the mechanism shown in Figure 16. When the adaptor was reversed and the unlock button was pressed, the locking bar securing the lower part of the surgical tool and the locking bar of the adaptor would retract, thereby allowing the surgical tool to be moved into the adaptor. As the adaptor advanced, the tension in the spring would cause the locking bar to return, thereby securing the surgical tool to the adaptor [Figure 16].

**FIGURE 17:**
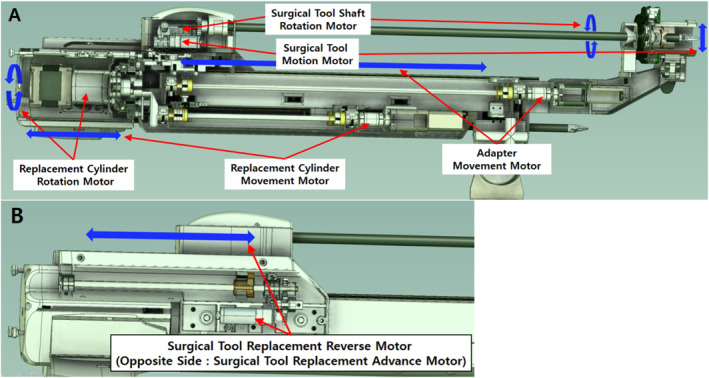
Arrangement of the surgical robot arm control motor. (The blue arrows indicate the direction of movement achieved by each motor). (A) Arrangement of surgical tool control motor, replacement cylinder control motor, and adaptor control motor. (B) Arrangement of surgical tool replacement motor (right side).

The drive system of the designed surgical robot was organised based on the motions required to drive the mechanism. By combining the tool replacement mechanism and motions of the tools required during actual surgery, the surgical robot should be able to perform a total of six motions. The tool replacement mechanism performs four motions: adaptor advancing and reversal, tool replacement advancing and reversal, replacement cylinder rotation, and replacement cylinder advancing and reversal. The actual surgery requires axial rotation of the surgical tool and tool motion (cutting, gripping, retracting, etc.); therefore, two additional motions were added. To facilitate control, we used one motor for each motion, with the exception of the advancing and reversal motions for tool replacement, which were achieved through pins pushing and pulling it on both sides; therefore, we separated the advancing and reversal motions, and configured the drive system with two motors. Consequently, we could control the entire drive of the surgical robot arm using a total of seven motors [Figure 17]. We established general requirements for motor selection, considering the roles of the seven motors to be used in the drive system [Table 1]. Motor No. 1 is the Replacement Cylinder Rotation Motor at the rear end of the robot arm, which rotates 120° to replace tools. Motor No. 2 is the surgical tool replacement advanced motor on the left side of the robot arm, which moves surgical tools from the replacement cylinder to the adaptor. Motor No. 3 is the surgical tool replacement reverse motor on the right side of the robot arm, which moves the surgical tool from the adaptor to the replacement cylinder. Motor No. 4 is the replacement cylinder movement motor located in the middle of the lower part of the robot arm, which moves the replacement cylinder forward and backward. Motor No. 5 is the adaptor movement motor on the front of the robot arm, which moves the tool adaptor forward and backward. Motor 6 is the surgical tool shaft rotation motor inside the surgical tool component, which rotates the surgical tool. Motor 7 is the surgical tool motion motor inside the surgical tool component, which grasps or releases the surgical tool.

**TABLE 1:** Motor selection requirements.

No.	Movement	Function	Specifications
1	Replacement cylinder rotation	120° rotation (both directions)	Gear reduction ratio: 1/51 Size: ∅42∗68.2mm Weight: 364g Rotational speed: 180 RPM
2	Surgical tool installation	Move the surgical tool from the cylinder to the adaptor, Return to the original position after performing the function	Gear reduction ratio: 1/36 Size: ∅8∗34.6mm Weight: 21.1 g Rotational speed: 333 RPM
3	Surgical tool removal	Move the surgical tool from the adaptor to the cylinder, Return to the original position after performing the function	Gear reduction ratio: 1/36 Size: ∅8∗34.6mm Weight: 21.1 g Rotational speed: 333 RPM
4	Replacement cylinder forward and backward movement	Replacement cylinder forward and backward movement	Gear reduction ratio: 1/44 Size: ∅16∗65mm Weight: 90.6 g Rotational speed: 278 RPM
5	Surgical tool forward and backward movement	Tool adaptor forward and backward movement	Gear reduction ratio: 1/54 Size: ∅16∗58.9mm Weight: 72g Rotational speed: 863 RPM
6	Surgical tool shaft rotation	Surgical tool rotation (both directions)	Gear reduction ratio: 1/64 Size: ∅8∗37.1mm Weight: 21.1 g Rotational speed: 333 RPM
7	Surgical tool motion	Surgical tool movement (1 degree of freedom per tool)	Gear reduction ratio: 1/64 Size: ∅8∗37.1mm Weight: 21.1 g Rotational speed: 333 RPM

**FIGURE 18:**
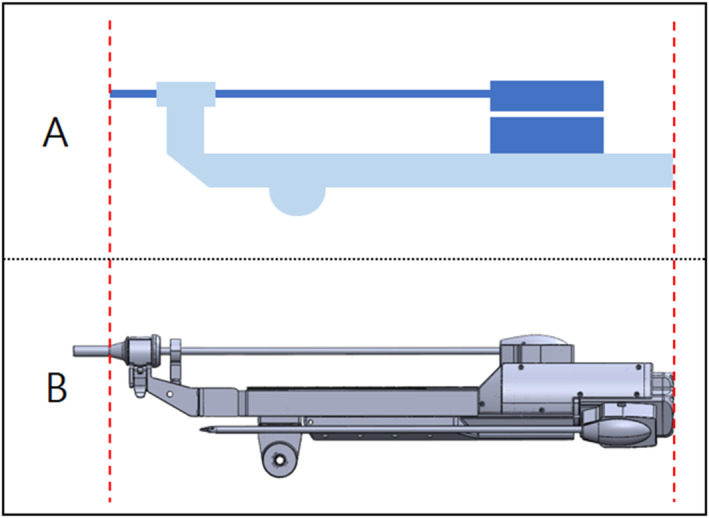
Comparison of the overall size of the existing surgical robot tool holder and the automatic tool replacement surgical robot tool holder proposed in the paper (front‐to‐back length) (A) size model of existing surgical robot. (B) Size model of the surgical robot proposed in the paper.

**FIGURE 19:**
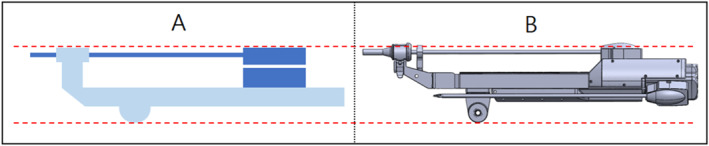
Comparison of the overall size of the existing surgical robot tool holder and the automatic tool replacement surgical robot tool holder proposed in the paper (vertical length) (A) size model of existing surgical robot. (B) Size model of the surgical robot proposed in the paper.

In addition, in order to eliminate the possibility of interference with other devices during actual surgery, we minimised the space required for tool replacement, and as a result, we were able to design a system similar in size to existing surgical robot systems [Figures 18, 19].

### 3.2 Dynamics Simulation for Drive Analysis

**FIGURE 20:**
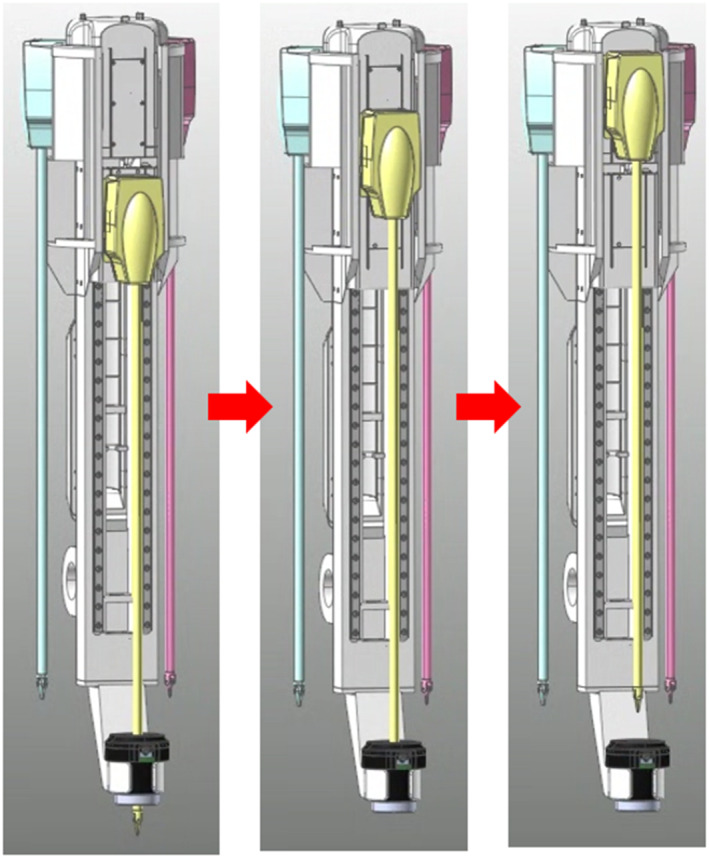
Simulation of surgical tool reversal.

**FIGURE 21:**
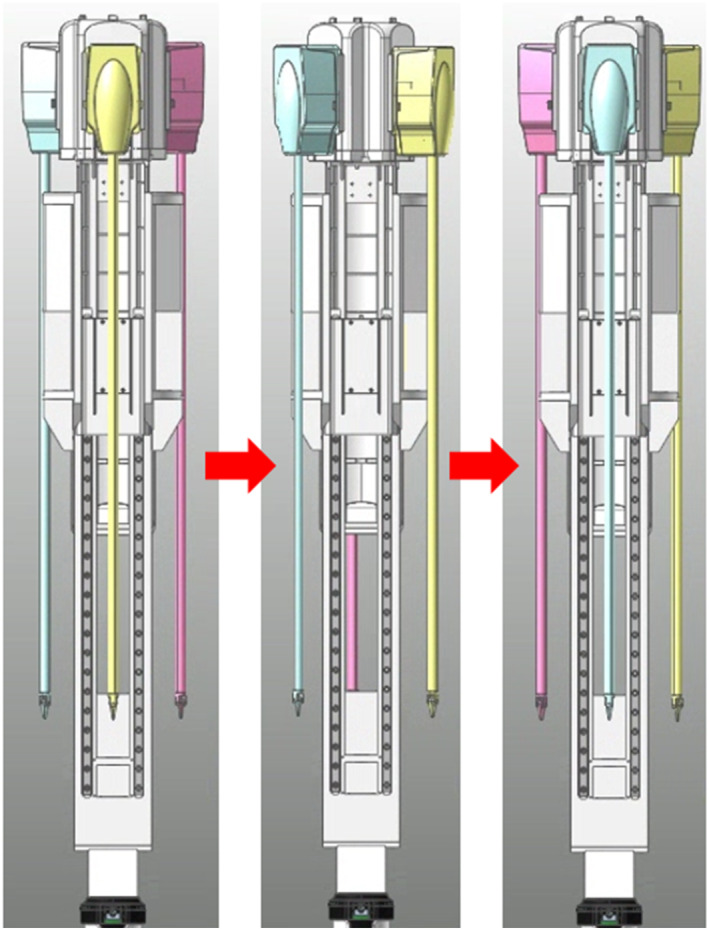
Simulation of replacement cylinder rotation.

**FIGURE 22:**
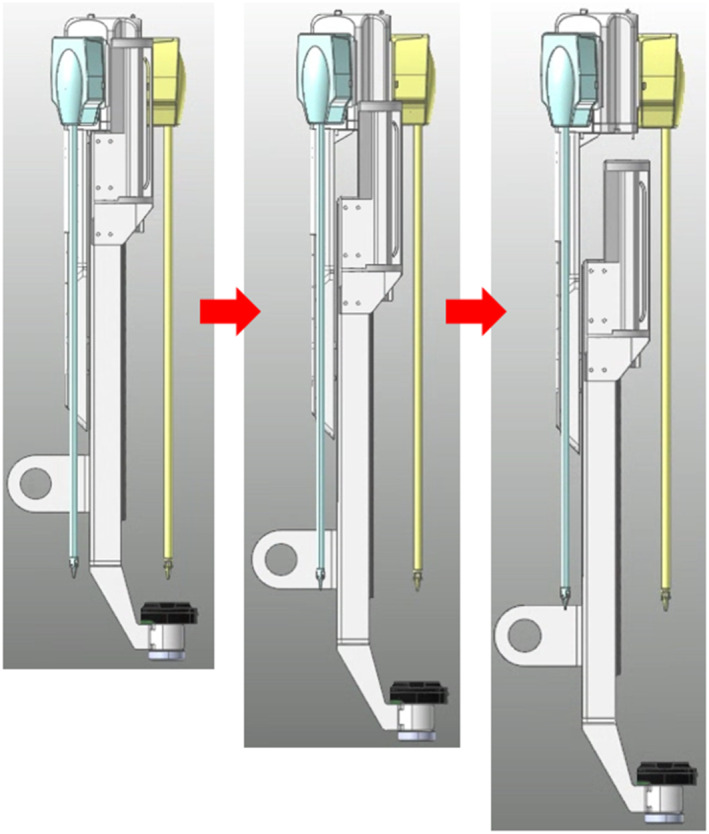
Simulation of replacement cylinder reversal.

**FIGURE 23:**
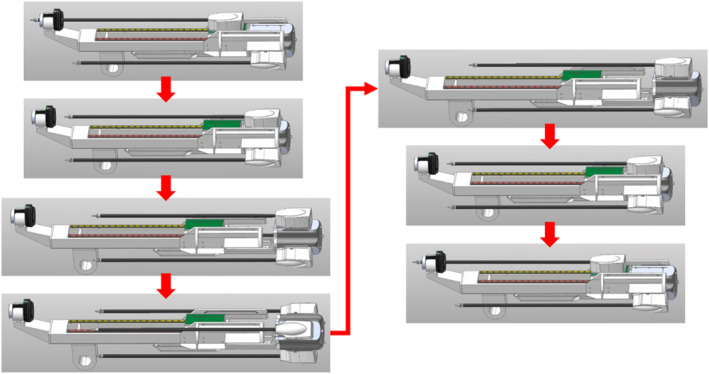
Dynamic simulation of all stages of the tool replacement process.

Before manufacturing the actual model, we created a 3D model and verified it with the dynamics software RecurDyn (V9R3, FunctionBay Inc., South Korea) [Figures 20, 21, 22]. Checking for interference between the drive devices and confirming the reaction force values at each stage of the process were carried out by analysing all stages of the tool replacement process using a dynamic simulation model [Figure 23]. Through the above process, we verified that there was no interference among the tools in the main motions for the automatic tool replacement of the surgical robot, namely advancing and reversal of the adaptor, advancing and reversal of the surgical tool, rotation of the replacement cylinder, and advancing and reversal of the replacement cylinder. We estimated the force required for the tool replacement by deriving the reaction force value from each movement and incorporated it in the manufacturing.

**FIGURE 24:**
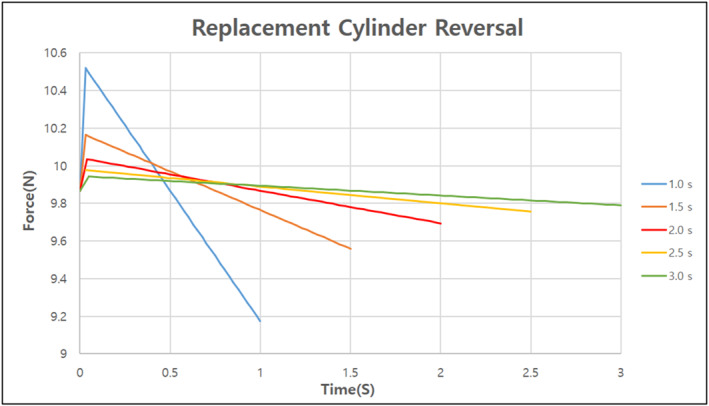
Reaction force per operating time generated when the replacement cylinder reverses.

**FIGURE 25:**
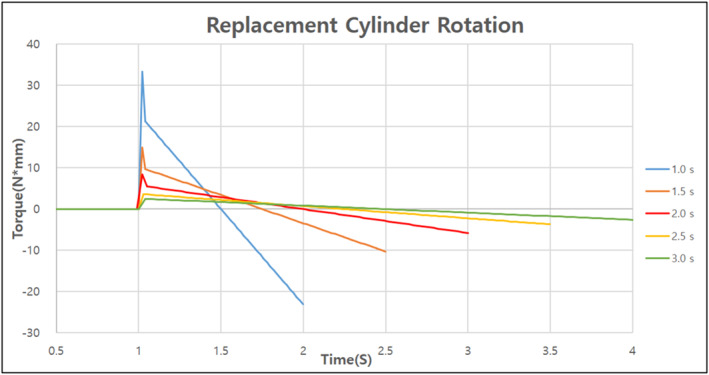
Reaction force per operating time generated when the replacement cylinder rotates.

**FIGURE 26:**
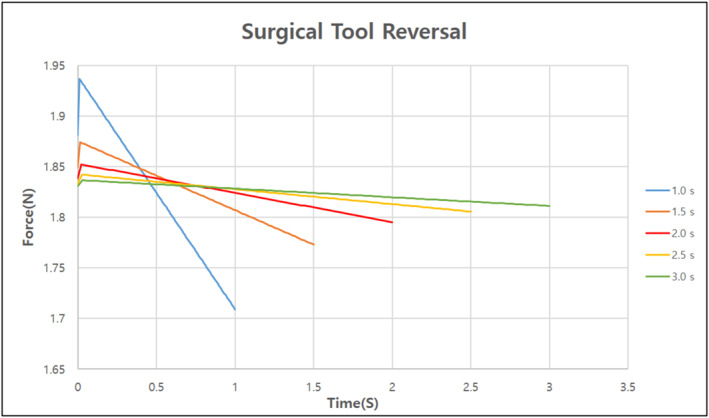
Reaction force per operating time generated when the surgical tool reverses.

The final model created through the aforementioned design process required a total of 27 s to replace the surgical tools automatically. However, for the surgical process to proceed smoothly, the time required to replace the surgical tools needs to be minimised. Using the dynamics software RecurDyn, the optimal replacement time was determined by identifying the speed at which the maximum and minimum reaction forces did not change significantly when the speed of the surgical robot was increased for each replacement [Figures 24, 25, 26][Tables 2, 3, 4].

**TABLE 2:** Variation in reaction force with replacement cylinder reversal speed (unit: N).

Operating time [s]	1	1.5	2	2.5	3
Maximum force [N]	10.520	10.164	10.034	9.976	9.942
Minimum force [N]	9.173	9.559	9.694	9.756	9.790

**TABLE 3:** Variation in reaction force with replacement cylinder rotation speed (unit: N‐mm).

Operating time [s]	1	1.5	2	2.5	3
Maximum torque [N‐mm]	33.293	14.925	8.431	3.606	2.497
Minimum torque [N‐mm]	−23.107	−10.268	−5.775	−3.696	−2.567

**TABLE 4:** Variation in reaction force with surgical tool reversal speed (unit: N).

Operating time [s]	1	1.5	2	2.5	3
Maximum force [N]	1.937	1.874	1.852	1.842	1.837
Minimum force [N]	1.709	1.773	1.795	1.806	1.811

In the plot showing the change in the reaction force according to the reversal speed of the replacement cylinder, the maximum and minimum values of the reaction force did not change significantly after 2 s of operation; therefore, we shortened the operation time from 7 to 3 s. The rotational speed of the replacement cylinder was fixed at 3 s because we found that the maximum and minimum values of the reaction force would change more widely if it was lowered below 3 s. In the case of the surgical tool reversal, we determined that the reaction force did not change significantly as a function of the drive time, and the part was lightweight and did not require significant motor drive; therefore, we shortened the drive time from 8 to 3 s. In conclusion, the final model could automatically change the surgical tools within 15 s, which we consider to be the clinically necessary speed for surgery.

### 3.3 Build and Validate Robotic Systems

**FIGURE 27:**
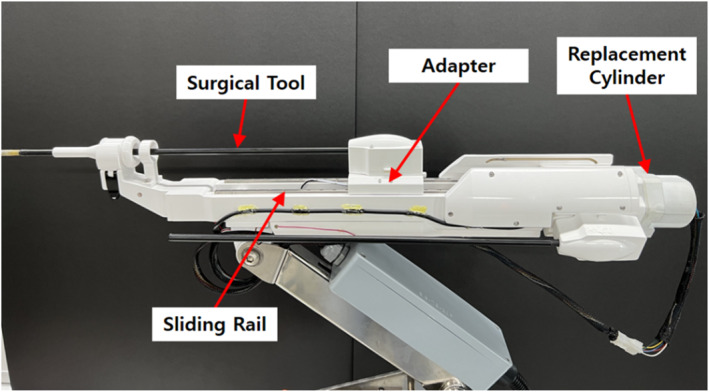
Structure of the surgical robot arm based on the final design.

**FIGURE 28:**
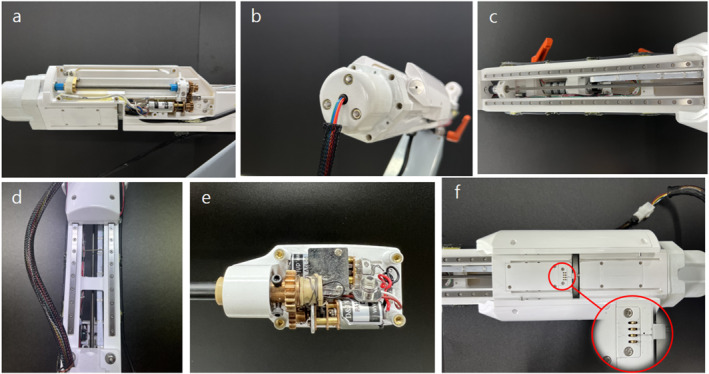
Detailed design of surgical robot tool replacement system. (A) atructure of surgical tool replacement reversal motor (right side), (B) replacement cylinder rotation part (with slip ring), (C) sliding rail drive part for adaptor advancing and reversal movements, (D) sliding rail drive part for replacement cylinder advancing and reversal movements (bottom view), (E) surgical tool drive part, (F) power and control signal communication between the surgical instrument and slider.

In this study, we built a robotic arm equipped with an automatic surgical tool replacement system that reflected our design [Figure 27]. The robotic arm is composed of a surgical tool, adaptor, replacement cylinder, sliding rail, and body, as shown in Figure 27; it performs the same role as described above. The arm is equipped with two surgical tools other than the one being used, thus making it possible to switch among three surgical tools. While a surgical tool is replaced, the adaptor is driven either by the replacement reversal motor and screw located on the right side or the replacement advancing motor and screw located on the left side [Figure 28a]. The cable used to power the motor is connected to the back of the replacement cylinder, and a slip ring is used to prevent interference with the connected power cable when the replacement cylinder rotates [Figure 28b]. The forward and backward movements of the adaptor during the tool change process are driven by a screw and motor located on the slider drive part [Figure 28c], The forward and backward movements of the tool change cylinder are driven by a sliding rail and motor at the bottom [Figure 28d].

In this study, the rotation, opening, and closing of the surgical tool (forceps) were realised using a surgical tool drive motor to operate the surgical tool [Figure 28e], and a contact‐type power signal connection terminal was used to transmit the signal from the controller to the surgical tool motor [Figure 28f].

**FIGURE 29:**
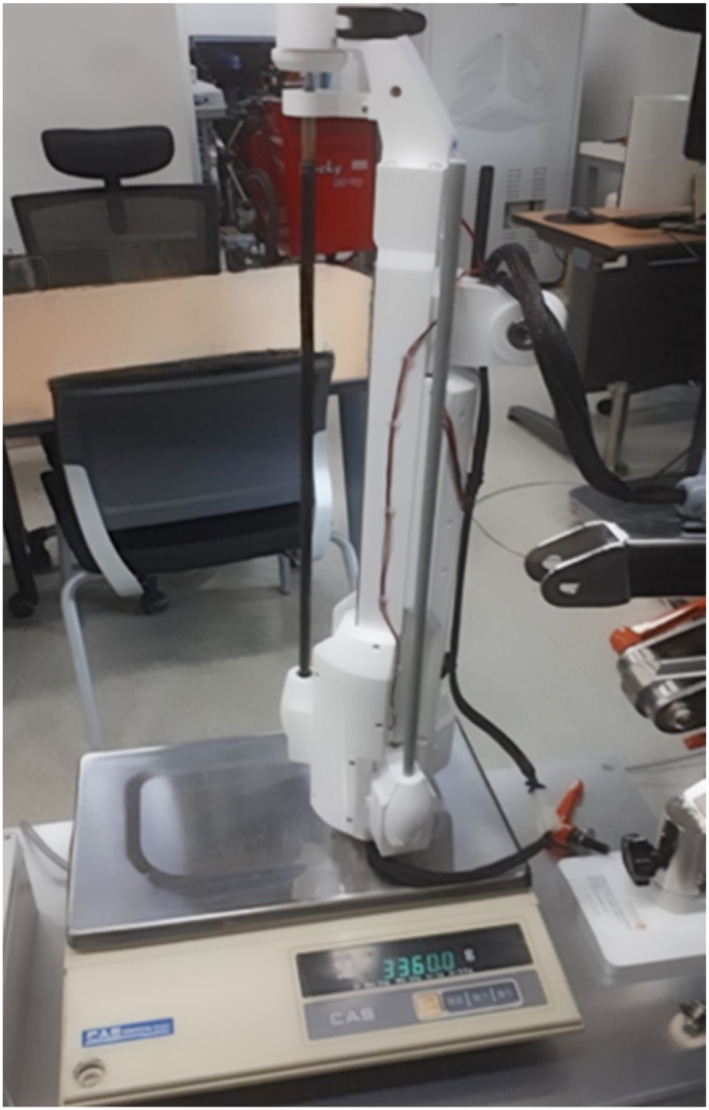
Mass measurement of all components of the robot arm (3.36 kg).

After completing the production, including all engineering elements, we measured the mass of the robot arm. The total mass of the robot arm was measured at 3.36 kg, achieving our target mass of less than 4 kg [Figure 29].

**FIGURE 30:**
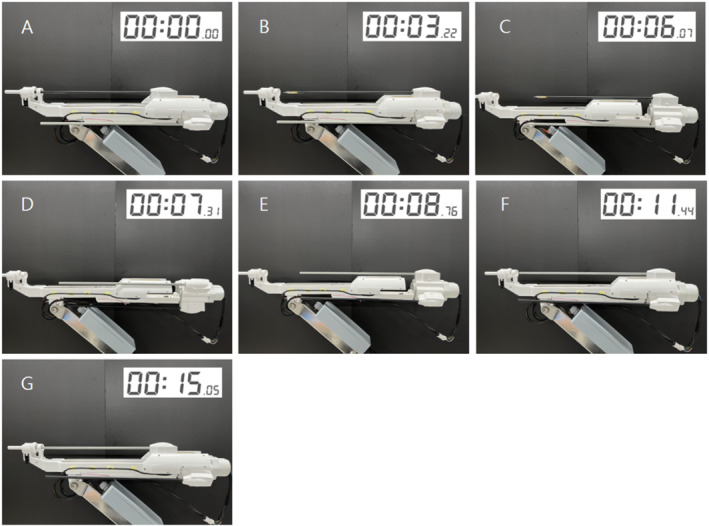
Automatic surgical instrument replacement process and actual time required for each instrument replacement step. (A) Initial state. (B) Moving surgical tool from adaptor to replacement cylinder. (C) Replacement cylinder reversed. (D) Replacement cylinder rotated. (E) Surgical tool selected. (F) Replacement cylinder advanced. (G) Surgical tool moved from replacement cylinder to adaptor. (Replacement completed).

Next, the performance of the automated surgical tool replacement system was tested [Figure 30]. The system was driven by removing the tool from the adaptor, replacing it, and remounting it. Sequences A–B in Figure 30 comprise the first step in Figure 14, which describe the final mechanism: tool reversal (moving from the adaptor to replacement cylinder). Sequences B–E comprise the second step in Figure 14, which include the reversal and rotation of the replacement cylinder; this is followed by E–F, which constitute the third step in Figure 14, which is the advancement of the replacement cylinder and insertion of the surgical tool into the adaptor. The driving test showed that the developed system could perform the tool replacement process in 15 s without interference between the devices [Figure 30].

## 4 Discussion

This study was conducted with the purpose of implementing solo surgery in existing laparoscopic robotic surgery. Therefore, it is essential to consider the clinical requirements related to laparoscopic surgical robots, and Chapter 2 clearly defines these requirements and driving performance. We proposed a development process for the target mechanism and a method for confirming clinical safety, and finally presented a mechanism capable of implementing the required conditions and quantitative performance targets. In Chapter 3, we used the multi‐body dynamics software RecurDyn to verify the drive and interference between mechanisms, derived the optimal drive speed for reducing the replacement speed and lightening the drive motor, and completed the design of the surgical instrument replacement device. Additionally, the designed surgical instrument replacement device was actually manufactured to verify drive performance metrics such as the relative size of the instrument components, instrument mass, and replacement time, thereby confirming the suitability of the developed mechanism.

The surgical robot automatic tool replacement system developed in this study will contribute to the establishment of a solo‐surgery system by substituting the tool replacement process, which is currently performed by an assistant. Furthermore, this device is capable of maintaining the posture during the tool replacement, which is not achieved by many of the current surgical robot replacement devices. These characteristics of surgical robots, compared with those of the existing tool replacement devices, make it easier for surgeons to perform surgeries. As a result, the automatic tool replacement system for surgical robots developed in this study will solve the problems of shortage of surgical personnel while providing the same proficiency as the surgical assistants.

## 5 Conclusion

In this study, we investigated and developed an automatic tool replacement mechanism for surgical tools used in a laparoscopic surgical robotic system. In existing laparoscopic robots, surgical tools are replaced manually by a surgical assistant; however, developing a robotic system that automates this process for solo surgeries is essential. Therefore, we designed and manufactured such a mechanism for development and practical implementation, and verified the same. We accomplished a tool replacement mechanism that does not require much space compared with the existing surgical robots. To drive the developed mechanism, we designed the selection and placement of motors, drive transmission mechanisms, and detailed mechanisms and tools for installing, removing, fixing, and releasing the surgical tools. The final model was built as a prototype, and a control system (motors and actuators) was implemented to drive the same. Operational tests were performed to prove that the automatic tool replacement mechanism is feasible in practice.

The purpose of the automatic surgical tool replacement mechanism developed in this study is to create a solo‐surgery system for surgeons. Therefore, this automatic surgical tool replacement mechanism is expected to play a significant role in replicating the actions of a surgical assistant for tool replacement. Future research on the automated replacement of surgical tools will focus on its automatic control, using artificial intelligence for predicting the surgeon's intentions. However, in terms of the robot mechanism that drives the actual instruments, research should be directed towards minimising the moment generated by the instruments and improving the drive speed for a smooth surgery. Additionally, developing a mechanism suitable for real‐world application scenarios and for realising a lightweight and easy control of the mechanical parts that implement it is necessary.

## Author Contributions

**Daehwan Ko:** conceptualisation, methodology, visualisation, investigation, data curation, writing – original draft preparation. **Yeonkyoung Kim:** investigation, data curation, formal analysis. **Hongseok Lim:** methodology, project administration, writing – review and editing. **Sungmin Kim:** supervision, resources, funding acquisition, writing – review and editing.

## Ethics Statement

The authors have nothing to report.

## Conflicts of Interest

The authors declare no conflicts of interest.

## Permission

The materials from other sources have been reproduced in accordance with the licence terms at the address below. Licence: https://creativecommons.org/licenses/by/4.0.

## Data Availability

Research data are not shared.
